# Measuring Global Credibility with Application to Local Sequence Alignment

**DOI:** 10.1371/journal.pcbi.1000077

**Published:** 2008-05-16

**Authors:** Bobbie-Jo M. Webb-Robertson, Lee Ann McCue, Charles E. Lawrence

**Affiliations:** 1 Computational Biology and Bioinformatics, Pacific Northwest National Laboratory, Richland, Washington, United States of America; 2 Department of Applied Mathematics and the Center of Computational Molecular Biology, Brown University, Providence, Rhode Island, United States of America; University of Chicago, United States of America

## Abstract

Computational biology is replete with high-dimensional (high-D) discrete prediction and inference problems, including sequence alignment, RNA structure prediction, phylogenetic inference, motif finding, prediction of pathways, and model selection problems in statistical genetics. Even though prediction and inference in these settings are uncertain, little attention has been focused on the development of global measures of uncertainty. Regardless of the procedure employed to produce a prediction, when a procedure delivers a single answer, that answer is a point estimate selected from the solution ensemble, the set of all possible solutions. For high-D discrete space, these ensembles are immense, and thus there is considerable uncertainty. We recommend the use of Bayesian credibility limits to describe this uncertainty, where a (1−*α*)%, 0≤*α*≤1, credibility limit is the minimum Hamming distance radius of a hyper-sphere containing (1−*α*)% of the posterior distribution. Because sequence alignment is arguably the most extensively used procedure in computational biology, we employ it here to make these general concepts more concrete. The maximum similarity estimator (i.e., the alignment that maximizes the likelihood) and the centroid estimator (i.e., the alignment that minimizes the mean Hamming distance from the posterior weighted ensemble of alignments) are used to demonstrate the application of Bayesian credibility limits to alignment estimators. Application of Bayesian credibility limits to the alignment of 20 human/rodent orthologous sequence pairs and 125 orthologous sequence pairs from six *Shewanella* species shows that credibility limits of the alignments of promoter sequences of these species vary widely, and that centroid alignments dependably have tighter credibility limits than traditional maximum similarity alignments.

## Author Summary

Sequence alignment is the cornerstone capability used by a multitude of computational biology applications, such as phylogeny reconstruction and identification of common regulatory mechanisms. Sequence alignment methods typically seek a high-scoring alignment between a pair of sequences, and assign a statistical significance to this single alignment. However, because a single alignment of two (or more) sequences is a point estimate, it may not be representative of the entire set (ensemble) of possible alignments of those sequences; thus, there may be considerable uncertainty associated with any one alignment among an immense ensemble of possibilities. To address the uncertainty of a proposed alignment, we used a Bayesian probabilistic approach to assess an alignment's reliability in the context of the entire ensemble of possible alignments. Our approach performs a global assessment of the degree to which the members of the ensemble depart from a selected alignment, thereby determining a credibility limit. In an evaluation of the popular maximum similarity alignment and the centroid alignment (i.e., the alignment that is in the center of the posterior distribution of alignments), we find that the centroid yields tighter credibility limits (on average) than the maximum similarity alignment. Beyond the usual interest in putting error limits on point estimates, our findings of substantial variability in credibility limits of alignments argue for wider adoption of these limits, so the degree of error is delineated prior to the subsequent use of the alignments.

## Introduction

The study of genomics, and much of computational molecular biology, is about the inference or prediction of discrete, high-dimensional (high-D) unobserved variables, based on observed data. For example, in RNA secondary structure prediction, the challenge is to select a specific set of base pairs from a combinatorially large collection, as a prediction of the secondary structure of an RNA polymer, given its sequence. Similarly, in pathway inference, the challenge is to select a set of graph edges to connect genes or their products (nodes) from a combinatorially large collection of possible edge sets, based on gene expression or other data. Model selection problems for studying diseases stemming from mutlifactorial inheritance are becoming increasing common in the post-genome era. In these studies, the ultimate goal is to identify the combinations of genes responsible for inheritance components of disease etiology based on genetic and/or other post-genome data. In motif finding, the challenge is to select a single member of a large ensemble of possible combinations of motif sites in a set of sequences. Procedures that select the single best scoring solution, such as maximum similarity, maximum likelihood, maximum *a-posteriori* (MAP), or minimum free energy, dominate nearly all of these problems.

Sequence alignment is a typical example and is arguably the most important high-D discrete prediction problem for biology. Because it is the cornerstone capability used by a multitude of computational biology applications, we employ sequence alignment to make these general concepts concrete. Sequence alignment methods commonly focus on identifying the highest scoring alignment between two sequences, and assessing the statistical significance of this alignment [1]–[7]. Thus, alignment algorithms, heuristic [5], [8]–[10] (http://www.ncbi.nlm.nih.gov/BLAST/) and optimization [11] (http://fasta.bioch.virginia.edu/fasta_www2/) alike, typically report the selected alignment, and a statistical score that assesses how likely an alignment with a score as good or better could have emerged by chance, under a specified null distribution (commonly an *E*-value). While methods that assign the significance of alignments under a null distribution have been well studied, assessments of the uncertainty of a proposed alignment, defining the confidence in this alignment and assessing its overall reliability, have received considerably less attention.

Regardless of the alignment procedure employed, when a single alignment is chosen for the comparison of two (or more) sequences, it is a point estimate (or estimating alignment) selected from a large ensemble of all possible alignments. For example, two sequences of length *m* and *n* have 
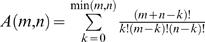
 possible local alignments, where *k* represents the number of matches in the alignment [11],[12]. This number grows rapidly with the length of the sequences being aligned; for example, two small sequences of only length 20 generate over 10^29^ possible local alignments. The question addressed here is: How, based on the available data, should we articulate the overall uncertainty of a selected estimating alignment (how well does it represent the large ensemble of possible solutions), and thus assess the reliability of this alignment?

The traditional approach to address the reliability of a single alignment is to evaluate the optimal alignment in the context of a set of near-optimal alignments. Near-optimal or suboptimal alignment analysis involves evaluating residue alignment consistency over the set of defined near-optimal alignments [12]–[17]. Specifically, the reliability of an alignment position (*i*,*j*) is assessed by comparing the score of the optimal alignment to the score of this alignment under the constraint that positions *i* and *j* do not align [14],[15]. More advanced methods have been proposed that determine reliability measures between residues aligned to both residues and gaps [17]. An alternative to computing near-optimal alignments, involving a single model that assigns probabilities to a specific residue pair, such as a pair Hidden Markov Model [7],[18],[19], can be derived and used to assess the reliability of individual aligned pairs.

With this in mind, these near-optimal alignment and model-based methods have offered significant improvements in reliability for tasks such as structural alignment. However, these methods are focused on delineating the reliability/uncertainty of the individual components of an estimated alignment, not the reliability of an estimated alignment in the context of the entire alignment space. There are methods to assess the accuracy of an alignment in the prediction of a ground-truth standard such as an alignment based on crystal structures [7], [18], [20]–[22]. But our focus here is on assessment of the reliability of an alignment based on its own characteristics, rather than the assessment of its accuracy in predicting an established reference. Toward this end, we describe a procedure for global assessment of the degree to which the members of the ensemble may depart from a selected estimate.

The introduction of probabilistic alignment methods [23]–[26] established the notion of sequence alignment as an inference procedure. For example, optimization-based alignment routines often search for the single alignment that is most probable among all those in the entire space of alignments. It is not surprising, given the immense size of the alignment space, that the most probable alignments, and thus all individual alignments, often have very small probabilities. This finding raises three questions:

1. In discrete spaces, how strongly does the available data recommend a single chosen estimate?
2. When the data provide weak evidence for any single estimate, what criteria can be used to judge the credibility of an estimate, and what are reasonable limits in the degree of variation within the ensemble from this estimate that are consistent with the data?
3. How can we identify the single estimate that best represents the ensemble of alignments and that is consistent with the data?

We suggest the following answers to these questions:

1. The strength of the recommendation of the data for any specific estimate is equal to its posterior probability under the assumed probabilistic model.
2. A credibility limit is the radius of the smallest hyper-sphere around a proposed estimate that contains a specified proportion of the probability mass of the posterior distribution, where the radius is measured by the number of elements by which two solutions differ. The size of this limit characterizes an estimate's credibility.
3. The estimate with the minimum credibility limit best represents the ensemble.

To address these questions and test our proposed answers, we employ a Bayesian probabilistic approach. In the Methods section, we review some concepts on probabilistic alignments and distance measures, and then consider the distribution of the distances of the alignments in an ensemble from a proposed estimating alignment, including the quantiles and expected value of this distribution. We use the quantiles to identify credibility limits. The identification of credibility limits begs the question: What procedures can be developed to identify alignments with tight credibility limits? In an effort to achieve this goal, we employ statistical decision theory to find an estimation procedure that identifies the estimates with the minimum average distance from the posterior weighted ensemble; that is, the centroid. Centroid estimators, which were recently described by Carvalho and Lawrence [27], look promising to yield tight credibility limits because they minimize an average Hamming distance. Furthermore, we show that since popular procedures that select an estimate because it scores better than any other single solution (e.g., maximum likelihood, maximum similarity, maximum *a-posteriori* Viterbi solutions) are optimal under a zero/one-loss function, there is no principled reason to expect them to have tight credibility limits and, thus, to have high credibility. Below we compare the credibility limits for centroid alignments to those for maximum similarity alignments.

## Methods

A statistical model that yields a probability distribution over an ensemble of solutions is essential for the characterization of uncertainty. Specifically, we are interested in using the data, in combination with any parameters that have been specified, to assign “posterior” probabilities to the members of the ensemble. We call these posterior probabilities because they are assigned after considering the implications of the data, the posterior weighted ensemble. Because in high-D settings it is often impossible to characterize the entire immense ensemble of solutions, it is common practice to employ representative samples from the posterior distributions to draw inferences or make predictions [28].

### Probabilistic Alignment

A probabilistic alignment model from which samples can be drawn can be described as follows. An alignment describes a set of aligned residues and associated insertion and deletion events. For a pair of sequences, 
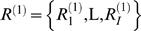
 and 
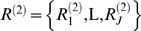
, let *A* be a matrix that characterizes an alignment whose (*i*,*j*)-entry is defined as:
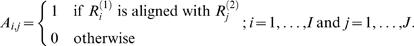
Without loss of generality, let *I*≤*J*. Because a residue cannot align with more than one other residue, two constraints must be satisfied, 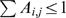 and 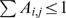. In addition, the alignment co-linearity constraint requires that *A_i,j_*+*A_k,l_*≤1, *i*≤*k*, *l*≤*j*. Let Θ be a matrix of residue pair similarities, such as one of the BLOSUM [29] or PAM [30] scoring matrices, and let Λ = (*λ* *_o_*, *λ* *_e_*) be the probability of opening and extending a gap, respectively.

Most sequence alignment methods optimize an objective function that can be described, based on a probabilistic model, as a log-likelihood [31],[32]. In traditional (frequentist) statistics, only the observed data, here *R* ^(1)^ and *R* ^(2)^, are seen as random variables, and the remaining terms are deterministic variables with perhaps unknown values. In maximum likelihood estimation, the values of these unknowns, which maximize the likelihood, are the maximum likelihood estimates. Typically, the user must set specific parameter values for the scoring matrix Θ^0^ and gap probabilities Λ^0^ to find the most probable alignment A* over all possible alignments:
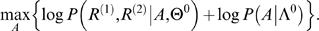
(1)This alignment is guaranteed to be the alignment that has the largest probability over all possible alignments, and with appropriate re-parameterization, it can also be shown to be the maximum similarity (MS) alignment [19].

To capture the entire alignment space in a probabilistic manner, the problem of alignment can be formulated as a Bayesian inference problem [19],[23],[26]. The Bayesian Algorithm for Local Sequence Alignment (BALSA) [24] describes such a probability model, the full joint distribution of all alignments, as the product of the likelihood and priors:
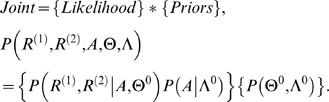
Recursion can be employed to marginalize (i.e., sum out) over all possible alignments to obtain the marginal probability of the data in the two sequences, given only the defined scoring matrix, Θ^0^, and gap penalties, Λ^0^:
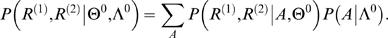
The required sums are completed in an analogous manner to the Smith-Waterman recursion by essentially replacing the maximum function with a summation. The alignment parameters Θ and Λ can also be defined as random variables and marginalized over using Markov chain Monte Carlo (MCMC) sampling methods. In this application, to mirror common alignment practice, a specific scoring matrix (PAM 110) and gap-penalty parameters (gap opening = −14 and gap extension = −2) were selected as generic parameters used by sequence alignment algorithms. Now the probability of any single alignment can be computed as a posterior probability using the following Bayes formula:
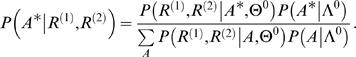
(2)Equation 2 is a ratio of the likelihood of the data and the alignment A* to the sum of these joint likelihoods over all alignments. It approaches a value of 1 when a single alignment dominates all others.

Given that the number of possible alignments for even small biopolymer sequences is immense, it is not feasible to calculate the probability of all alignments in a brute force manner. However, we can almost always use the recursive relationships that are fundamental to dynamic programming (DP) to draw guaranteed representative samples from the solution ensemble [19]. Because of the power of the recursions, such sampling procedures require no burn-in period to ensure that the samples are drawn from the equilibrium distribution, and these samples are independent of one another. Briefly, these algorithms use modified versions of the two fundamental steps of DP: the forward and back-trace recursions. In DP, the forward recursion finds the optimal value of the objective function (e.g., the best total alignment score) by using optimal solutions of subproblems to recursively build up to the best total score. In the sampling algorithm, we instead use an analogous recursion to build up to the sum over the entire ensemble of solutions. This sum finds the normalizing constant that assures that probabilities sum to one. In the back-trace step, instead of finding the solution that yields the optimal value of the objective function, we use an analogous recursion to sample solutions in proportion to their posterior probabilities. An important unappreciated fact is that for large ensembles, the accuracy of estimates based on a sample depends on the sample size only, and not on the size of the population [23]. Thus, a representative sample (i.e., a sample drawn in proportion to the probabilities of the unknowns) of even modest size, say 1000, can yield accurate estimates of unknowns, even if this sample is drawn from an ensemble of immense size. As we illustrate below, representative samples can be used to estimate credibility limits and define an ensemble centroid (EC) solution.

### Credibility Limits and Means Distance

In this section, we describe procedures for finding credibility limits and mean distances for the sequence alignment problem. We begin by examining the distribution function of the distances of the ensemble members from a proposed estimate. Basic to this perspective are two concepts: 1) given the available data, the solution space is inherently uncertain; and 2) a proposed estimate is a point estimate (i.e., a single member of the ensemble) that is intended to represent the entire ensemble [33].

A simple measure of the difference between two members of a discrete ensemble (e.g., two possible alignments of a pair of sequences) is the Hamming distance. For two alignments, *A* ^(*k*)^ and *A* ^(*m*)^, of a pair of sequences, *R* ^(1)^ and *R* ^(2)^, of length *I* and *J*, the Hamming distance is simply the number of aligned positions that differ between *A* ^(*k*)^ and *A* ^(*m*)^, *D*(*A* ^(*k*)^, *A* ^(*m*)^). For alignments, this distance is simply the sum of the differences in two binary matrices of size (*I*×*J*). When ensemble members are binary objects, the Hamming distances are also equal to distances on other scales [34]:
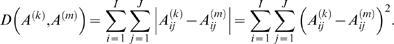
(3)Using the metric in Equation 3, the distance between any proposed estimating alignment and the ensemble of alignments can be computed regardless of how one selects the estimating alignment. In this report, we compare the results of using two different estimating alignments: *A^M^*, the MS alignment, and *A^C^*, the EC alignment.

Specifically, let *D_i_* = *D*(*A_i_*, *A^x^*) be the distance of the *i* ^th^ member, *A_i_*, of the ensemble from a proposed estimating alignment, *A^x^*, where X is a categorical variable indicating the estimator (*X*∈[*M*, *C*]). We then rank the ensemble members by their distances from *A^x^*, and let 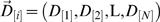 be the order statistics of these distances (i.e., the distances of the ensemble members from the estimating alignment) with the indices permuted to reflect their order in the distance ranking [35]. The distribution function of the distances is:
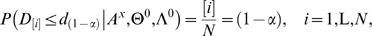
(4)where *d*_(1−α)_ is the (1−*α*)*^th^* quantile. Now the credibility limit at (1−*α*) is *d*_(1−*α*)_. While higher-order DP recursions can be used to obtain these limits, they can also be quite reasonably estimated from a representative sample of even modest size by the following algorithm [35]:

1. Draw a representative sample of size *p*, say *p* = 1000, elements by sampling from their posterior distribution, as illustrated for sequence alignment by Webb et al. [24].
2. Rank these alignments by their distance, *D_i_* = *D*(*A_i_*, *A^x^*), from the estimate *A^x^*.
3. Now *d̂*_(1−*α*)_, the (1−*α*)*^th^* quantile in this sample is our estimator of *d*_(1−*α*)_.

The expected value of *D_i_* is
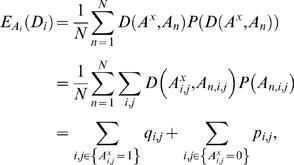
(5)where *A_n,i,j_* is 1 if *i* aligns with *j* in the *n* ^th^ member of the sample, and zero otherwise; *q_i,j_* is the marginal probability that *A_n,i,j_* = 0; and *p_i,j_* is the marginal probability that *A_n,i,j_* = 1. The required marginal probabilities can be estimated based on a sample, or when DP is available, they can be obtained using the forward- and back-trace algorithm described by Durbin et al. [19].

### Normalized Credibility Limit

**Figure 1:**
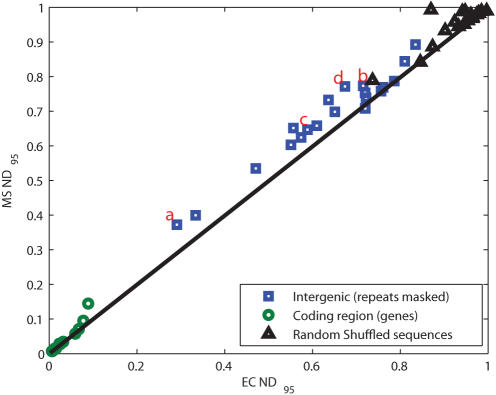
Plot of *ND*_95_ values for the EC versus the MS of 20 pairwise sequence alignments. The *ND*_95_ values associated with the 20 highly conserved gene sequences are represented as green circles. The sequence alignments that represent alignment of random, un-related, sequences are represented as black triangles. In blue squares are the *ND*_95_ values for the intergenic sequences upstream of the coding genes. The four example alignment *ND* distributions displayed in Figure 2 are indicated by a letter next to the corresponding square.

Hamming distances will, in general, be dependent on the lengths of the ensemble members. For example, in alignment, longer sequences will tend to return larger distances simply because the alignment matrix is larger. Thus, normalization is in order. For this normalization, we employ a normalization factor that uses maximum realized alignment lengths. Specifically, when calculating a credibility limit, the length of the estimating alignment (*LE*) is known, and the maximum length of an alignment in the ensemble is the length of the shorter of the two sequences (*I*). Thus, the maximum Hamming distances between an estimating alignment and the longest member of the ensemble is (*LE*+*I*). However, in our studies, we found that using this sum as a normalizing factor was misleading for cases in which the posterior space of alignments tended to be dominated by shorter local alignments. For example, the local alignments of the randomly shuffled sequences described in the Results section (see Figure 1) were dominated by short alignments. As a result, using (*LE*+*I*) as the normalizing constant in this case produced normalized distances that were not close to one, even when there were no base pairs in common between a sampled alignment and the estimating alignment. To adjust these differences, we used the length of the longest sampled alignment, *LS*, as the second term in our normalizing sum, and the normalizing distance between the estimating alignment *A^x^* and the *i^th^* alignment in the sample is 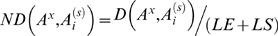 where *S* indicates the set of sampled alignments. Using this normalization factor yields normalizing distances with values between zero and one. A perfect match would yield an *ND* score of zero, and in the case where the longest sampled alignment has no base pairings in common with the estimating alignment, the *ND* score would be one. We define the credibility of the alignment at (1−α) to be *ND*_(1−*α*)_.

### Centroid Alignment Estimators

Maximum similarity alignments, and the associated Viterbi alignments, have been the dominant alignment procedures for decades. In these procedures, an alignment output is typically the single alignment that has the maximum probability over all possible alignments. However, having the largest probability does not indicate that it represents the alignment space described by the billions (or more) possible alignments, except in the unusual event that this single alignment alone has high probability. In fact, the most probable alignment, the MS alignment, often has very small probability. For example, in this study, the probabilities of the MS alignments ranged from 10^−37^ to 10^−249^ for the alignments of the human/rodent pairs of gene and promoter sequences. Because it is the most probable alignment for a pair of sequences, all other alignments for that pair can be no more probable than the MS alignment. Thus, from a Bayesian prospective, any individual alignment represents the data only weakly at best.

As Carvalho and Lawrence [27] point out, procedures that identify the single, highest scoring alignment are optimal under a zero/one loss function. Accordingly, after the highest scoring alignments have been identified, all other alignments have a penalty of one (i.e., are all equally unimportant); thus, if no single alignment has a high probability mass then the expected loss will be large. As a result, with zero/one loss there is no reason for the optimal alignment to be positioned near any other member of the ensemble of alignments, therefore failing to garner support from any other member of the ensemble.

In contrast, centroid alignments garner information from the complete ensemble of alignments, because these alignments minimize the expected Hamming distance from the complete posterior weighted ensemble of alignments. Centroid alignments correspond directly to the reliable alignments of Miyazawa with a cut off 0.5 [26]. Reliable alignments are further described by Durbin et al. [19] and are elaborated on by Holmes and Durbin [34]. Furthermore, because these alignments minimize the average Hamming distance, we expect that they may yield tighter credibility limits than MS alignments. The alignment that is the centroid of the entire ensemble of alignments is called the EC alignment. These alignments meet the exclusive pairing and colinearity constraints of the alignment problem, but they do not necessarily meet the common requirement that a gap in one sequence cannot be followed by a gap in the other sequence. We compare the credibility limits of MS alignments and EC alignments below.

## Results

To assess the credibility measures and estimators described above, we examine the local alignments of sequences from (1) 20 orthologous genes between human and rodent, and (2) 24 orthologous genes between six species of *Shewanella*. All sequence pairs were evaluated using BALSA [24] with a PAM 110 scoring matrix, gap penalties of −14 and −2 for opening and extending a gap, respectively, and a sample size of 1000 to compute the estimated alignment distributions, credibility limits, and EC alignments.

### Credibility Limits for Human/Rodent Pairs

The 20 orthologous genes for human/rodent are specifically up-regulated in human skeletal muscle tissue, and their upstream sequences have been used in previous studies to locate *cis*-regulatory modules [36]. The coding regions of the 20 human/rodent orthologous gene pairs were evaluated, as were the 20 sequence pairs that represent up to 3 kb of sequence upstream of the orthologous gene pairs. All sequence pairs were masked using RepeatMasker (http://www.repeatmasker.org/). For the local alignments of the 20 gene pairs and the 20 intergenic regions, we examined the credibility limits associated with two estimating alignments: the MS, and the EC. Specifically, we examined the 95% quantiles of the normalized distances (*ND*), computed based on the distances between these estimating alignments from the 1000 sampled alignments from the posterior alignment distribution. Figure 1 shows a scatter plot of the MS 95% credibility limits (MS *ND*_95_) versus the EC 95% credibility limits (EC *ND*_95_) for the local alignments of the genes and the intergenic regions. For contrast, the genes were randomly shuffled, and 95% credibility limits were defined for these non-related sequence pair alignments.

First, notice that the credibility limits for the gene sequence alignments are small, and the difference between the EC and MS is negligible. These genes are so highly conserved that the majority of the posterior distribution falls along a small set of paths with high probability, thus creating high correlation between the EC and MS. Alternatively, when the gene sequences are shuffled, the hyper-sphere surrounding 95% of the posterior distribution is very large because the probability of aligning any two residues is essentially random. This results in extremely large credibility limits with high deviation in the distance of the ensemble from the EC and MS. The intergenic regions are less conserved than the genes and, thus, are intermediate between these two extremes. Notice that the credibility limits are often surprisingly large, with normalized distances over 50% for 18 of the 20 MS alignments, and for 17 of the 20 EC alignments. This indicates that we have confidence in less than half the predicted aligned base pairs. As the plot shows, there is considerable variation in the credibility limits over the 20 examples when either the EC or MS limit is used. The credibility limits for the EC range from 29% of maximal to nearly 91%, while the MS limits range from 37% to almost 100% of maximal. This result highlights the need to report credibility limits for every sequence pair. We also see that for all but one of the sequence pairs, the MS credibility limits are greater than those for the EC. Furthermore, for 11 of the 20 upstream sequence pairs, the MS credibility limits were more than 600 base pairs larger than EC credibility limits. Thus while the differences in Figure 1 look modest, the MS credibility limits are often hundreds of base pairs larger than those of the EC estimators.

**Figure 2:**
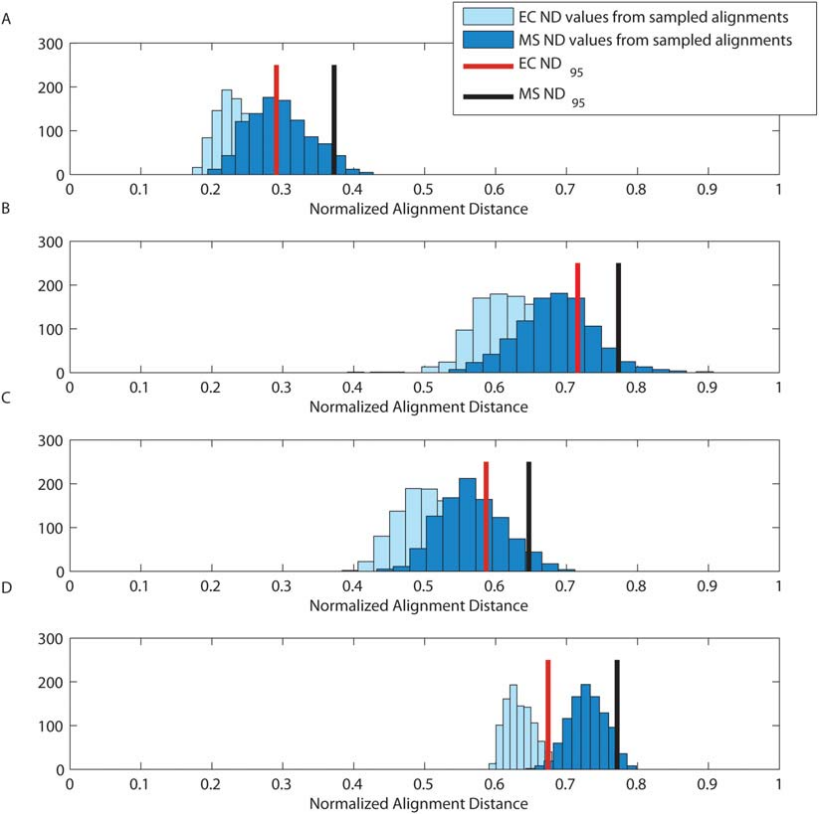
Histograms of the distances of the sampled alignments from the EC and MS. In (A) the centroid and optimal alignments are similar and represent the distribution well, but in (B) and (C), despite a similar centroid and optimal alignment, neither represent the overall alignment distribution. In (D) it is observed that the centroid and optimal deviate significantly from each other, and that the centroid is a much better representation of the alignment space.

Taken together, the differences between the 20 MS normalized distances and 20 EC normalized distances in Figure 1 are significantly different (i.e., p<0.001, Wilcoxon Signed Rank test [37]). To offer further insight, we chose four alignments from the 20 to examine in more detail (Table 1); the results for all 20 pairs are in Table S1. In Figure 2, we show histograms of the distance of the 1000 sampled alignments from the two estimating alignments (MS, EC); in addition, the 95% quantile (*ND*_95_) for the EC and MS are shown as bars, and the values are given in Table 1. As Figure 1 indicates, pair (A) has the tightest credibility limits of all the promoter sequences. These tighter limits are a reflection of the fact that the ensemble of alignments is relatively close to the estimators; the 95^th^ percentile alignment differs from the EC estimator by 270 of a possible 1556 base pairs that could potentially differ (*ND*_95_ = 0.29), while the MS is about 20% larger with an *ND*_95_ = 0.37. Of the 20 promoter sequence pairs, there are 11 in which the two credibility limits are markedly different (i.e., by more than 0.05). Figure 2D is another illustration of the characteristics of these 11 pairs for which the MS credibility limits are substantially larger than those of the EC, although for pair (D) the distance distributions have very little overlap, as well as large credibility limits. Figure 2C is representative of the remaining nine pairs, in which the posterior surface is quite flat, and the two credibility limits differ by less than 0.05. For the sequence pair shown in Figure 2C, the credibility limits for both estimators are large. Because the EC alignment is the nearest alignment to the mean [34], the large size of this limit for the EC alignment indicates that the alignments in the posterior distribution are widely dispersed over the ensemble. Also notice that in (B) and (C), the two distributions overlap substantially and have high *ND*_95_ values; for example, the alignment in Figure 2B shows a *ND*_95_ = 0.72 for the EC, and *ND*_95_ = 0.77 for the MS alignment. Because the centroid estimator is the closest feasible alignment to the mean, for this sequence pair the mean and the mode are close, as is typical of symmetric distributions [27].

**Table 1:** Gene, *ND*_95_, P-Quantile information on examples highlighted in Figures 1 and 2.

	RefSeq Gene Identifier		*ND*_95_	
	Human	Rodent	EC	MS
(A)	NM_001885.1	NM_012935.2	0.291	0.373
(B)	NM_000080.2	NM_009603.1	0.716	0.773
(C)	NM_001042.2	NM_012751.1	0.587	0.647
(D)	NM_003186.3	NM_011526.4	0.674	0.771

### Credibility Limits for *Shewanella*

**Figure 3:**
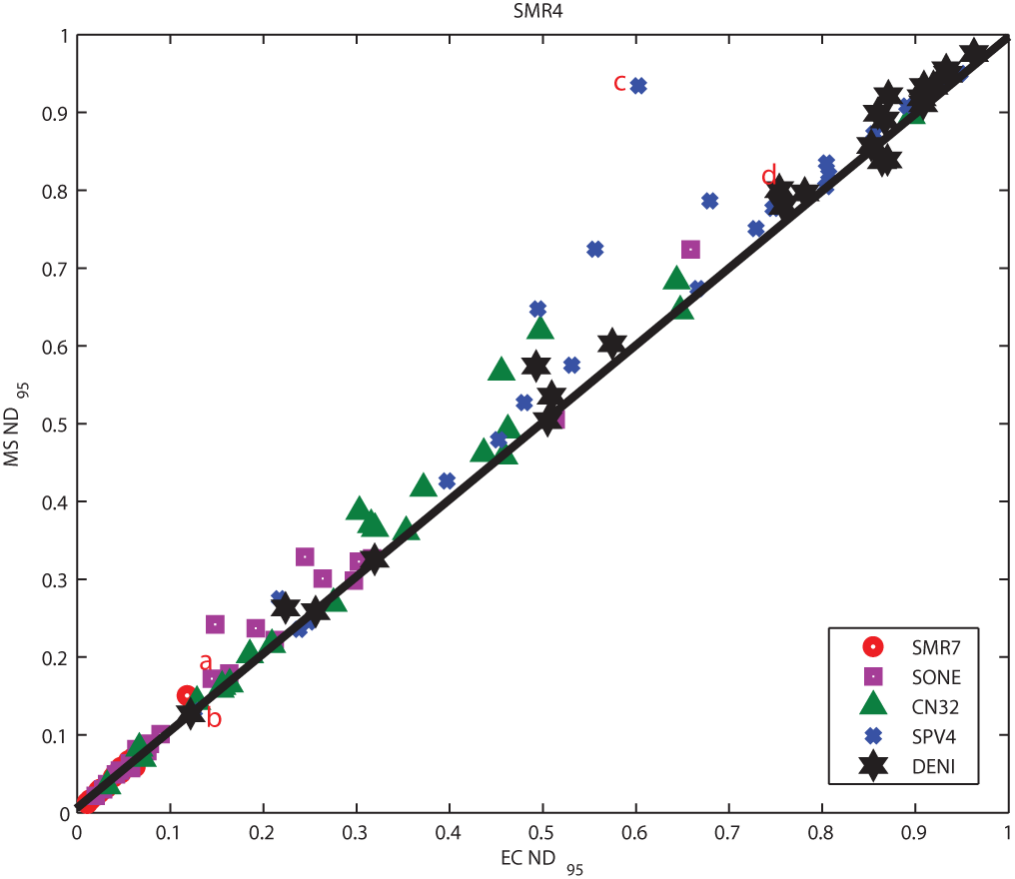
Plot of *ND*_95_ values for the EC versus the MS of 120 pairwise sequence alignments (24 comparisons for each of the five species in the legend to SMR4). The four example alignment *ND* distributions displayed in Figure 4 are indicated by a letter next to the corresponding symbol.

We also examined the credibility limits for the MS and EC estimators for local alignments of orthologous pairs of intergenic regions (up to 500 bp upstream of orthologous genes) from six species of *Shewanella* for which full genome sequence data are available: 1) *S. denitrificans* OS217 (DENI), 2) *S. loihica* PV-4 (SPV4), 3) *S. oneidensis* MR-1 (SONE), 4) *S. putrefaciens* CN-32 (CN32), 5) *Shewanella* sp. MR-4 (SMR4), and 6) *Shewanella* sp. MR-7 (SMR7). We chose SMR4 as our base species, aligning orthologous sequences from each of the other five to the region from SMR4. Starting with SMR4, the species in order of increasing evolutionary distance are SMR4>SMR7>SONE>CN32>SPV4∼DENI. As before, we examined the 95% quantiles of the normalized distances, computed based on the distances between the estimating alignments and the sampled ensemble of alignments drawn from the posterior alignment distribution. Figure 3 shows a scatter plot of the MS *ND*_95_ versus the EC *ND*_95_ values for each of 24 randomly selected orthologous regions, for the pairwise comparison of SMR4 to each of the five species at varying evolutionary distances (120 total comparisons).

The two species SMR4 and SMR7 are very closely related, having been isolated from samples taken at different depths (5 m and 60 m, respectively) from a single location (latitude and longitude) in the Black Sea [38]. Thus, it is not surprising that even the intergenic regions are highly conserved and that the EC and MS exhibit tight credibility limits. Among the comparisons to species at increasing evolutionary distance, we observe increasing credibility limits. In fact, for many of the SMR4-DENI sequence pairs, the credibility limits are no better than expected for randomly shuffled sequence. While, on average, the credibility limits of a pair of species increase with increasing evolutionary distance, the figure also shows that the credibility limits of the alignments for a given pair of species vary greatly. For example, even though the credibility limits of most SMR4-DENI pairs are large (>0.8), there are sequence pairs from these two species that have credibility limits <0.3. The fact that there is wide variability in credibility limits for all of these pairs of species, except SMR4-SMR7, highlights the importance of assessing the reliability (credibility limits) of nearly all alignments. For example, there is a pair of SMR4-CN32 sequences whose alignment is very reliable (EC *ND*_95_ and MS *ND*_95_<0.05), but there are also three pairs whose alignments cannot be trusted (EC *ND*_95_ and MS *ND*_95_>0.6), and the remainder are scattered over the full range in between.

**Figure 4:**
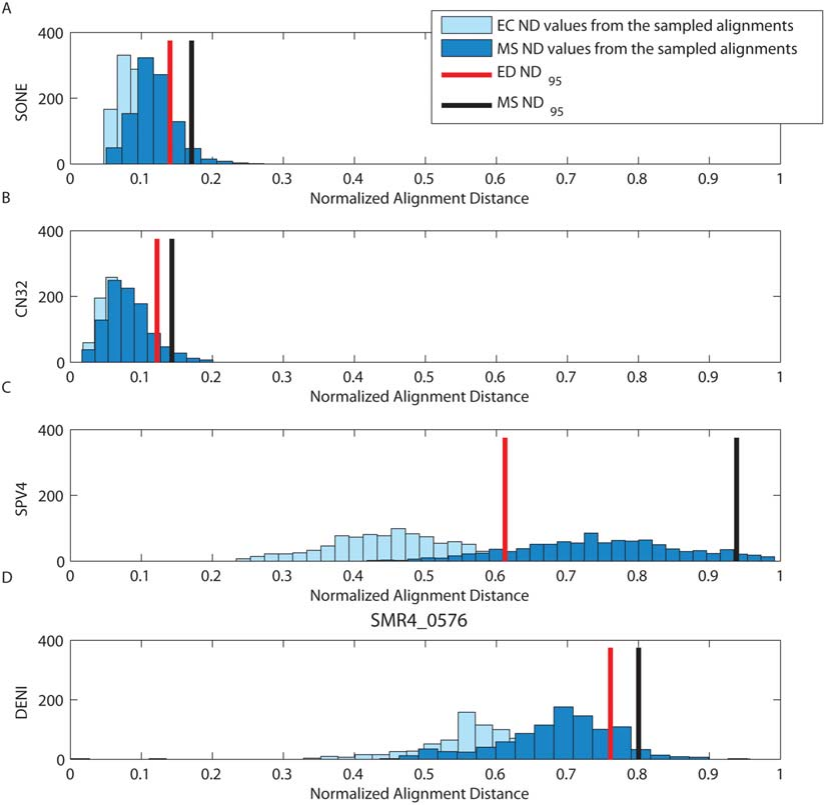
Histograms of the distances of the sampled alignments from the EC and MS for the intergenic regions upstream of the gene SMR4_0576. SMR4_0576 alignment distribution with its orthologous sequence from (A) SONE, (B) CN32, (C) SPV4, and (D) DENI.

**Figure 5:**
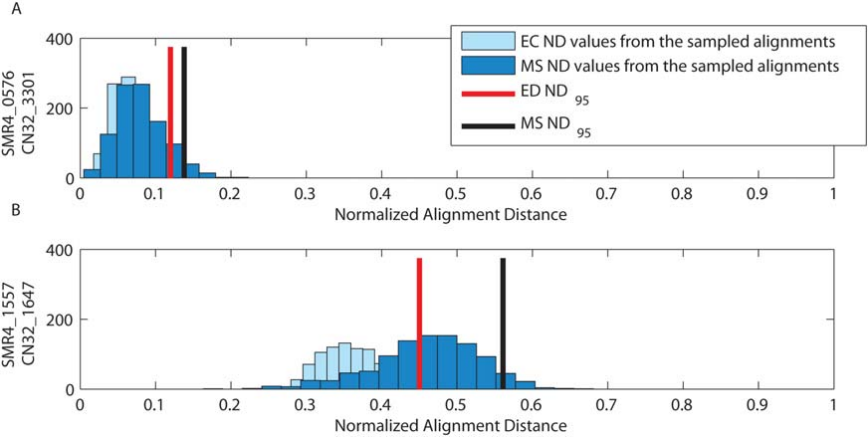
Histograms of the distances of the sampled alignments from the EC and MS for the intergenic regions upstream of orthologous genes from SMR4 and CN32. (A) Alignment distribution for the regions upstream of the orthologous genes SMR4_0576 and CN32_3301 and (B) alignment distribution for the orthologous regions upstream of the arginine decarboxylase (speA) genes SMR4_1557 and CN32_1647.

We further evaluated the findings shown in Figure 3 in the context of a single gene's orthologous upstream sequences. Often in evaluating promoter sequences across species it is unknown *a priori* which sequences it would be most beneficial to align. The tight credibility limits shown in Figure 4A and 4B indicate that when evaluating the promoter region of SMR4_0576, we would have confidence in the alignments with the orthologous region from SONE and CN32 (also with SRM7, data not shown). This is not the case for the orthologous regions from SPV4 and DENI. The high *ND*_95_ values for the EC and MS alignments indicate that alignment of SPV4 or DENI sequences would not contribute to a meaningful evaluation of the SMR4_0576 promoter region. Unfortunately, not all alignments of promoter regions from SMR4 with the promoter sequences of orthologous genes in SONE and CN32 are reliable. For example, as Figure 5 shows, the posterior distribution of the alignments of the SMR4_ 1557 promoter region with its CN32 ortholog is substantially more widespread and variable than the posterior distribution of alignments for the promoter region of SMR4_0576 with its orthologous region in CN32.

These findings of large differences in the reliability of alignments within species pairs have had a substantial practical impact on our studies of phylogenetic motif finding using these *Shewanella* species. Specifically, alignment of orthologous promoters can substantially increase the power of motif finding, if the alignments can be trusted. However, the findings shown in Figure 5 indicate that reliance on a single genome-wide measure of species distances is very frequently insufficient to assure that alignments of promoters from species pairs can be trusted. Thus, we are using credibility limits on a gene-by-gene and species-by-species basis to make decisions about which alignments can be trusted.

### Centroid Alignment Heat Map

**Figure 6:**
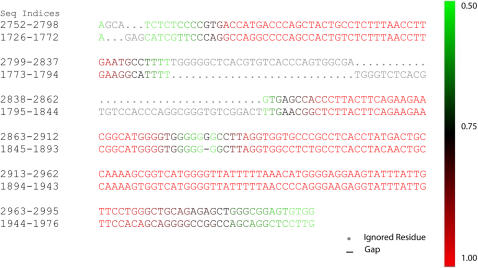
Heat-map alignment representation of the EC. Sequence indices are given on the left and the color gradient associated with aligned residue probabilities is given on the right. Sequence regions that have no aligned pairs with a probability greater than 0.5 are ignored by the alignment, grayed out, and aligned with a dot to differentiate these from insertion/deletion events that utilize a dash.

The use of heat maps or other means to visually illustrate confidence in the individual alignment of individual pairs of bases must accommodate a different feature for centroid alignments. Specifically, EC alignments have a feature not present in standard alignments, in that they allow stretches of sequence in the middle of an alignment to remain unaligned in a manner analogous to those regions at the ends of local alignments. That is, a residue in one sequence that cannot be *reliably* aligned with any single residue in the other sequence is excluded from the centroid alignment. Aligning any such residues to any bases in the other sequence would only increase the average distance of the centroid alignment from the posterior distribution of alignments. In addition, with probabilistic alignment, we return marginal probabilities of all residue pairs. Therefore, to display all the features of this alignment, we employ 1) a traditional dash to represent gaps, 2) a dot to represent residues that cannot be reliably aligned and are thus ignored in the alignment, and 3) a gradient color scheme (i.e., a heat map) to show the base pair alignment probabilities, where red indicates high probability for that residue pair, green indicates probabilities nearing 50%, and the ignored region is grayed out to further differentiate those residues for which the variability in alignments is too great to permit marginal pair probabilities of 0.5 or greater. Figure 6 gives an example of the heat map alignment display for a human/rodent intergenic sequence pair (the region upstream of the MYL2 gene). The red-to-green coloring of aligned regions allows quick distinction of areas of alignment of high versus low confidence.

## Discussion

Because prediction and estimation involve making inferences about unknown quantities based on the available data, they are inevitably uncertain. Thus, when a specific value is reported as a point estimate, it is common in many fields to simultaneously report a confidence limit or a credibility limit, which is the Bayesian analog. Such limits are all too often absent in computational biology. Here, to promote their broader adoption, we describe a method for estimating credibility limits and illustrate these concepts using sequence alignment. These credibility limits are derived from the empirical distribution function of the Hamming distance from the estimator to the members of the ensemble of solutions, or more accurately, a representative sample of the ensemble of solutions. The 95% credibility limit of a proposed estimate describes the posterior distribution by indicating the normalized Hamming distance containing 95% of the probability mass of the posterior distribution. The existence of these limits begs the question: What estimation procedure will yield tight credibility limits? We advocate the use of recently developed centroid estimators that minimize the expected Hamming distance to address this question.

While it is reasonable to expect centroid estimators to produce tighter credibility limits, it is not a guaranteed product of this procedure, because the centroid is the estimator that minimizes the average differences from the posterior ensemble, while the credibility limits are based on a quantile. Nevertheless, our finding of tighter credibility limits for EC alignments compared to MS alignments should come as no surprise, since the well-known zero/one loss risk associated with the latter estimators provides no principled reason to expect that such estimators will be near the center of the posterior distribution of alignments. On the other hand, centroid alignments, which are the alignment nearest to the multivariate mean of the posterior distribution, are centered in the posterior distribution [27].

### Performance

Our findings of 1) high variability in the credibility limits in the alignments of promoter sequences of 20 human/rodent sequence pairs and 2) similar high variability among 4 of the 5 pairs of *Shewanella* species highlight the need for assessing the overall reliability of sequence alignments. Without such limits, there is little to distinguish alignments that vary greatly from one another in their reliability. Furthermore, our findings indicate that centroid estimators have promising potential to improve sequence alignment. For example, for over half of the human/rodent non-coding sequence pairs (each of ∼3000 bases) in our sample, the EC and MS alignments differ by more than 600 base pairs, and similar relative differences are observed in *Shewanella* alignments. While we report here on the credibility of nucleotide sequence alignments, they are equally applicable and valuable for protein sequence alignments.

In some discrete high-D inference problems, the posterior ensemble of solutions may not only be asymmetric, but also it may be multimodal, as has been reported for RNA secondary structures [39]. Since, in such a case no single point estimate can reasonably represent the posterior ensemble, class-specific estimates, with one for each distinct class, will be required. In these cases, samples associated with each class can be used to find credibility limits for the class estimates, and the overall credibility limits around these class-specific estimates can be identified based on distances to the nearest class estimate.

As mentioned above, the probabilistic model used is a Smith-Waterman recursive DP algorithm whose Viterbi alignment corresponded exactly to the MS alignment reported here. Thus, differences in credibility limits reported here are solely the result of the differences in the estimation procedures. In addition, the alignment that minimizes expected Hamming distance loss and also follows the requirement concerning adjacent gaps in the two sequences are available using a DP algorithm [19],[34]. However this alignment can only increase the average Hamming distance above that of the centroid.

**Figure 7:**
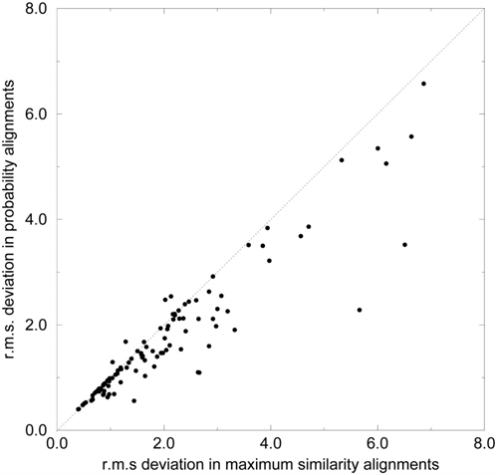
Root mean squared (r.m.s) deviation between the EC and MS for X-ray crystal structure data [26]. This scatter plot demonstrates that probability-based alignments (e.g., EC) typically have higher similarity with structural alignments than MS alignments.

While we believe this evidence supports reconsideration of the maximum scoring alignment paradigm, stronger evidence for reconsideration has been in the literature for over a decade. In 1995, Miyazawa [26] was the first to report what we now call centroid alignments [27]. In addition to his very insightful development of reliable alignments, he showed that these alignments are superior, using x-ray crystal structures of proteins as ground truth. Figure 7 (reproduced from Miyazawa's work [26], with permission of the author and Oxford Journals) shows that structural predictions based on reliable (centroid) alignments quite consistently produce lower root mean squared deviations than those based on maximum similarity alignments. Thus, from a practical biological prospective, there is already clear evidence in the literature that centroid alignments can be applied with advantage in the prediction of protein structures.

### Time Complexity

We also note that the time complexity of algorithms for obtaining centroid alignments and credibility limits is not different from those of more traditional optimization based methods. When recursions can be employed to obtain optimal solutions via DP, analogous recursions are frequently available for associated probabilistic models, and stochastic back-trace procedures can be employed to draw samples from the posterior ensemble of solutions [19]. In general, the time complexity for drawing these samples will be the same as that of the associated DP algorithm, and is set by the forward step of these algorithms. For example, in local sequence alignment, the most computationally intensive step is the forward-recursive step. For two sequences of length *n* and *m*, the time complexity is *O*(n*m) for both the optimization and Bayesian algorithms. Running times to obtain credibility limits in a recursive setting will generally be longer than times required to obtain optimal estimates because a back-trace step must be executed only once to obtain the optimal, while it must be employed multiple times to draw samples. However, this sampling will not generally greatly increase overall running times, because back-trace recursions are usually of a lower time complexity than their forward steps. For example, for local alignments the time complexity of the back-trace recursions is only *O*(min(*n*,*m*)). For problems not open to recursive solutions, MCMC algorithms are commonly employed, using procedures like simulated annealing. Credibility limits and centroids also can be obtained using MCMC sampling with run times that may be less than those for optimizations [27].

### Caveats

Some caveats are appropriate. In settings in which uncertainty is low, such as shown for the alignments of coding regions of human/rodent sequence pairs in Figure 1 and the promoter sequence pairs of very closely related species like *Shewanella* sp. MR-4 and MR-7 in Figure 3, credibility limits will likely be tight and not vary greatly among examples. Nevertheless, it would be reassuring to document this low variability by reporting credibility limits. While we have given principled arguments supporting our belief that centroid solutions should dependably have tighter credibility limits than optimization estimators, this advantage cannot be guaranteed. However, this trend was observed in both the human/rodent pairs and the *Shewanella* pairs. In our on-going work with *Shewanella*, we have found 1329 orthologous genes that were present in all six species and computed the 95% credibility limits for both the MS and EC, for all the promoters from SMR4 aligned with the orthologous sequences from each of the remaining 5 strains. The EC *ND*_95_ credibility limits were smaller than the MS *ND*_95_ limits in 6078 (91.55%) of these 6645 sequence pairs (i.e., p<1e-100, Wilcoxon Signed Rank test [37]).

In our comparison of centroid alignments to MS alignments, we focused on the alignment of individual pairs of sequences. However, we did not address how these two estimators would compare if we had available multiple pairs of sequences all drawn from a model with a single common “true” alignment. In the context of sequence alignment, such a situation would not be observed in nature because we know of no families of biological sequence pairs for which one can be confident that sequence pairs within this family all follow the same “true” alignment. For example, even for sequence pairs drawn from orthologous regions from clearly related species, alignments are likely to differ. This same absence of replicates, all of which are sampled from the same “true” value of the unknown, is expected for many, but not necessarily all, high-D discrete biological inference problems. Even when obtaining a large number of such biological replicates is possible in principle, such as a large number of biological replicates in a microarray study, obtaining them in practice is often prohibitively expensive. However, with advances in technology, this limitation may be overcome. When a substantial number of such replicate observations are available, the asymptotic properties of maximum likelihood estimates, such as consistency and asymptotic unbiasedness, can be brought to bare. In such cases, as sample size increases, the MS estimator will approach the true value, and the bias will tend toward zero. This reduction in bias might well counter-balance the higher variability (high credibility limits) reported here for individual sequence pairs.

The findings reported in this paper are for pairwise alignments. When multiple alignments are employed, we expect credibility limits to narrow because of the increased size of the data sets; however, we caution that the alignment space grows rapidly with increasing sequences in an alignment. Therefore, these limits may or may not shrink as quickly as expected. Furthermore, it is important to keep in mind that the credibility limits reported here are sampling estimates of true 95% quantiles, but with samples of 1000 the error bars on these estimates are 95%±1.35%. All the estimates in this work are based on a local probabilistic alignment model. While local alignment is the most common procedure, other probabilistic alignment procedures, or local alignments with other parameter settings [25],[26], may give varying results. As is common practice, all alignments here are given for a fixed set of parameters. Alignment parameters also can be estimated from the data; perhaps with such an approach, credibility limits could be smaller and more consistent, although this may not be the case because uncertainty of the parameter estimates would be introduced into the procedure.

### Conclusions

Beyond the usual interest in putting error limits on point estimates, our findings of substantial variability in credibility limits of alignments argues for wider adoption of these limits, so that the degree of error is delineated prior to the subsequent use of the alignments. From a practical prospective, when credibility alignments are tight, those using these alignments in subsequent procedures can be confident in the input alignments and know the limited degree to which input alignment may vary. The absence of such limits may well lead to a false sense of confidence in subsequent findings, especially when credibility limits are wide, and/or seriously limit an investigator's ability to determine the source of difficulties or inconsistencies in subsequent procedures that depend on these unreliable alignments. In practice, knowing early in a study that alignments required for subsequent results are unreliable (i.e., have high credibility limits) might well lead an investigator to reconsider his/her plans. For example, in studies of phylogenetic tree reconstruction when it is known that input alignments are reliable, investigators' conclusions about phylogenetic relationships will be bolstered; whereas, prior knowledge that input alignments are unreliable will motivate serious investigators to revise their study design or, after the fact, permit reviewers to raise legitimate questions about the studies conclusions.

While the results presented here concern only sequence alignment, the procedures described are generally applicable to point estimates for high-D discrete spaces; this includes many major inference problems in computational biology, such as pathway prediction in systems biology, the prediction of phylogenetic trees, the reconstruction of ancestral states, the delineation of alternate splice forms, and prediction of RNA secondary structures. For any of these problems, the algorithm given in the Methods section “Credibility limits and means distance” can be employed to obtain *ND*_95_ values for any proposed estimate given a procedure for drawing samples from the posterior distribution. We caution that while the Hamming distance will be appropriate in many of these areas, it may not be as appropriate in some of these settings. Regardless of the distance measure used, the proposed procedure will return credibility limits for an estimator when a representative sample can be obtained. We believe the use of confidence or credibility limits is long overdue throughout the full spectrum of discrete high-D inference problems encountered in computational biology. These limits have a number of valuable uses, including gauging the degree by which solutions might depart from their estimated value, appraising the overall credibility of a prediction, and comparing the performance of alternative estimators in cases where a “gold standard” is not available.

## Supporting Information

Table S1 Gene, ND95, and P-Quantile information on all 20 sequence pairs.
(0.04 MB DOC)
