# Supplementary material for: Measuring Global Credibility with Application to Local Sequence Alignment
Source: PLoS Comput Biol. 2008 May 16;4(5):e1000077. doi: 10.1371/journal.pcbi.1000077 (PMC2367447; doi:10.1371/journal.pcbi.1000077)
Supplement: Table S1 — Gene, ND95, and P-Quantile information on all 20 sequence pairs. (0.04 MB DOC) [file pcbi.1000077.s001.doc]

**Table 1. Gene, *ND*_95_, P-Quantile information on all 20 sequence pairs**.

| **RefSeq Gene Identifier** | | ***ND*_95_** | |
| --- | --- | --- | --- |
| **Human** | **Rodent** | **EC** | **MS** |
| NM_000747.2 | NM_009601.3 | 0.334 | 0.339 |
| NM_005119.4 | NM_009604.3 | 0.835 | 0.892 |
| NM_000751.1 | NM_021600.2 | 0.551 | 0.603 |
| NM_000080.2 | NM_009603.1 | 0.716 | 0.773 |
| NM_005159.4 | NM_009608.3 | 0.471 | 0.535 |
| NM_001824.2 | NM_007710.2 | 0.786 | 0.787 |
| NM_001927.3 | NM_010043.1 | 0.610 | 0.658 |
| NM_001042.2 | NM_012751.1 | 0.587 | 0.647 |
| NM_002479.4 | NM_031189.1 | 0.637 | 0.733 |
| NM_002476.2 | NM_010858.4 | 0.756 | 0.757 |
| NM_003281.3 | NM_017184.1 | 0.557 | 0.651 |
| NM_001100.3 | NM_009606.2 | 0.651 | 0.698 |
| NM_001885.1 | NM_012935.2 | 0.291 | 0.373 |
| NM_005205.2 | NM_009943.2 | 0.761 | 0.769 |
| NM_000258.2 | NM_010859.2 | 0.720 | 0.753 |
| NM_000432.2 | NM_012605.1 | 0.722 | 0.738 |
| NM_005368.2 | NM_013593.2 | 0.720 | 0.708 |
| NM_000290.2 | NM_018870.2 | 0.810 | 0.844 |
| NM_003186.3 | NM_011526.4 | 0.674 | 0.771 |
| NM_006172.2 | NM_012612.1 | 0.574 | 0.624 |
